# Coronavirus (COVID-19) Fulminant Myopericarditis and Acute Respiratory Distress Syndrome (ARDS) in a Middle-Aged Male Patient

**DOI:** 10.7759/cureus.8808

**Published:** 2020-06-24

**Authors:** Hussain Hussain, Aya Fadel, Haidar Alwaeli, Victor Guardiola

**Affiliations:** 1 Internal Medicine, Cardiology Clinic, Pasadena, USA; 2 Internal Medicine, Florida International University, Hialeah Hospital, Miami, USA; 3 Medical Education and Simulation, Richmond University Medical Center, New York, USA; 4 Oncology, Baptist Health South Florida, Miami, USA

**Keywords:** coronavirus disease (covid-19), diffuse st elevation, myocarditis, sars-cov-2 (severe acute respiratory syndrome coronavirus -2), ards, myopericarditis

## Abstract

Myopericarditis remains a prominent infectious inflammatory disorder throughout a patient’s lifetime. Moreover, viral pathogens have been proven to be the leading contributors to myopericarditis in the pediatric and adult populations. Despite the current comprehensive knowledge of myocardial injury in viral and post-viral myopericarditis, the cellular and molecular mechanisms of SARS-CoV-2-induced myopericarditis are poorly understood. This report presents a case of coronavirus (COVID-19) fulminant myopericarditis and acute respiratory distress syndrome (ARDS) in a middle-aged male patient: a 51-year-old man with a history of hypertension who arrived to the emergency department with a dry cough, fatigue, dyspnea, and a fever. A real-time reverse transcriptase-polymerase chain reaction (RT-PCR) assay confirmed a diagnosis of COVID-19 infection, resulting in the patient’s admission to the airborne isolation unit for clinical observation. When his condition began to deteriorate, the patient was transferred to the cardiac care unit after electrocardiography detected cardiac injury, demonstrating diffuse ST-segment elevation. Laboratory evaluations revealed elevated troponin T and BNP, with an echocardiogram indicating global left ventricular hypokinesia and a reduced ejection fraction. The patient was treated with hydroxychloroquine, azithromycin, dobutamine, remdesivir, and ventilatory support. This specific case highlights the severity and complications that may arise as a direct result of COVID-19 infection.

## Introduction

Coronavirus disease 2019 (COVID-19), a viral respiratory disease of possible zoonotic origin that surfaced in 2019, is caused by severe acute respiratory syndrome coronavirus 2 (SARS-CoV-2) [1]. Patients with confirmed severe acute respiratory syndrome coronavirus SARS-CoV-2 infection can present with flu-like symptoms that may include a fever, lethargy, anosmia, ageusia, rhinorrhea, myalgia, a dry cough, a sore throat, and other nonspecific symptoms such as diarrhea or abdominal pain [1]. Severe complications of COVID-19 infection include pneumonia, respiratory failure from acute respiratory distress syndrome (ARDS), septic shock, and acute myopericarditis [1-2]. This report presents a middle-aged male patient diagnosed with COVID-19 who, unfortunately, did not survive, addressing the data from the patient’s admission until his expiration.

## Case presentation

Clinical history

A 51-year-old middle-aged Italian man with a history of hypertension presented to the emergency department with a dry cough, fatigue, dyspnea, and a fever. He denied having any travel history, chills, diaphoresis, chest pain, or change in bowel or urinary habits. He also reported having multiple episodes of epigastric pain and nausea that had partially improved with omeprazole treatment two days prior to hospitalization. Upon his arrival to the emergency department, a physical examination revealed a body temperature of 39.6 °C, a respiratory rate of 26 breaths/min, a blood pressure of 141/89 mmHg, a heart rate of 97 beats/min, and an oxygen saturation of 91% (>95%) while the patient was breathing ambient air. An arterial gas analysis indicated a pH of 7.44 (7.36-7.44), an oxygen partial pressure of 79 mmHg (75-100 mmHg), and a carbon dioxide partial pressure of 39 mmHg (35-45 mmHg). Auscultation of the chest uncovered bilateral wheezing and rhonchi. Agonal respiration and a symmetrical decrease in chest expansion were noted as well.

The complete blood count test findings were normal, except for lymphopenia (920 lymphocytes/microliter). Since pneumonia was suspected, the patient was started on oxygen therapy, continuous electronic vital signs monitoring, an acetaminophen intravenous (IV) drip, ceftriaxone, and vancomycin. A nasopharyngeal swab was collected and tested for severe acute respiratory syndrome (SARS) associated coronavirus using the reverse transcriptase polymerase chain reaction (RT-PCR) method, and a positive result was obtained. In coordination with emergency medical services, hospital leadership and staff decided to discontinue the patient’s antibiotic treatments. The patient was then transferred to the airborne isolation unit for clinical observation.

On the first night of admission, the patient’s condition continued to worsen, and he received a more comprehensive evaluation. His physical examination revealed a respiratory rate of 29 breaths/min, a blood pressure of 134/87 mmHg, a heart rate of 120 beats/min, and an oxygen saturation of 84%. The patient was started on continuous positive airway pressure and the antiviral drug remdesivir to improve his oxygen saturation and halt the progression of the disease.

**Figure 1:**
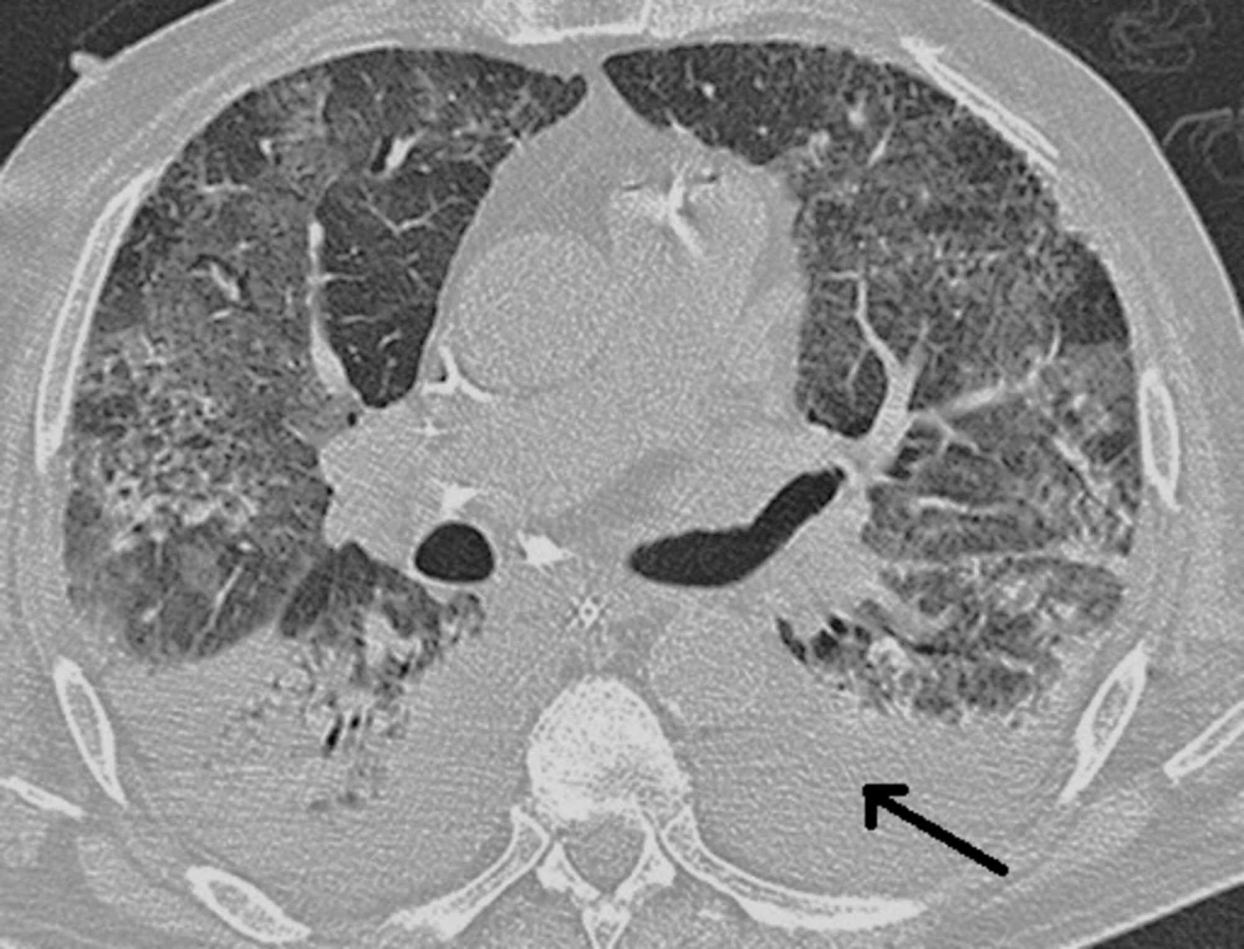
Chest CT scan without IV contrast of the patient with coronavirus infection establishing severe ARDS. CT: Computer Tomography; IV: Intravenous; ARDS: Acute Respiratory Distress Syndrome.

**Figure 2:**
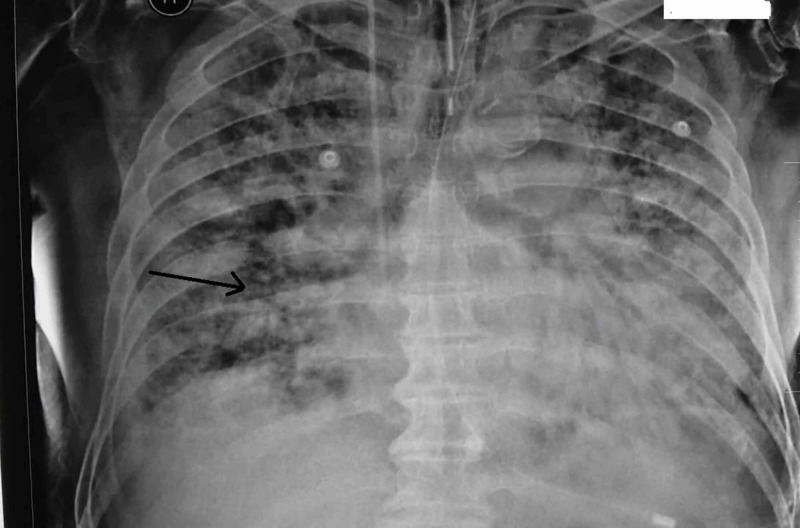
Chest X-ray PA view revealing a severe ARDS. PA: Posteroanterior; ARDS: Acute Respiratory Distress Syndrome.

During his second day of hospitalization, the patient remained febrile (a body temperature of 39.1 °C) and hypoxemic (an oxygen saturation of 81%) and required mechanical ventilation. A posteroanterior chest X-ray exposed bilateral and peripheral ground-glass and consolidative pulmonary opacities. These radiographic findings explain the progressive deterioration in the patient’s respiratory status and establish the diagnosis of ARDS (Figures 1, 2). Additionally, electrocardiography, troponin, and creatine kinase myocardial band (CK-MB) tests provided normal results.

The following day, the patient’s condition continued to deteriorate, and no improvements were noted. On examination, he appeared unwell: his vital signs revealed a body temperature of 37.8 °C, a blood pressure of 138/93 mmHg, a heart rate of 93 beats/min, and an oxygen saturation of 83% while he was on a ventilator. Additional medical therapies were initiated based on existing practice guidelines. The patient’s medications were reconciled, and hydroxychloroquine and azithromycin were added. No significant improvements were noted on the following day.

**Figure 3:**
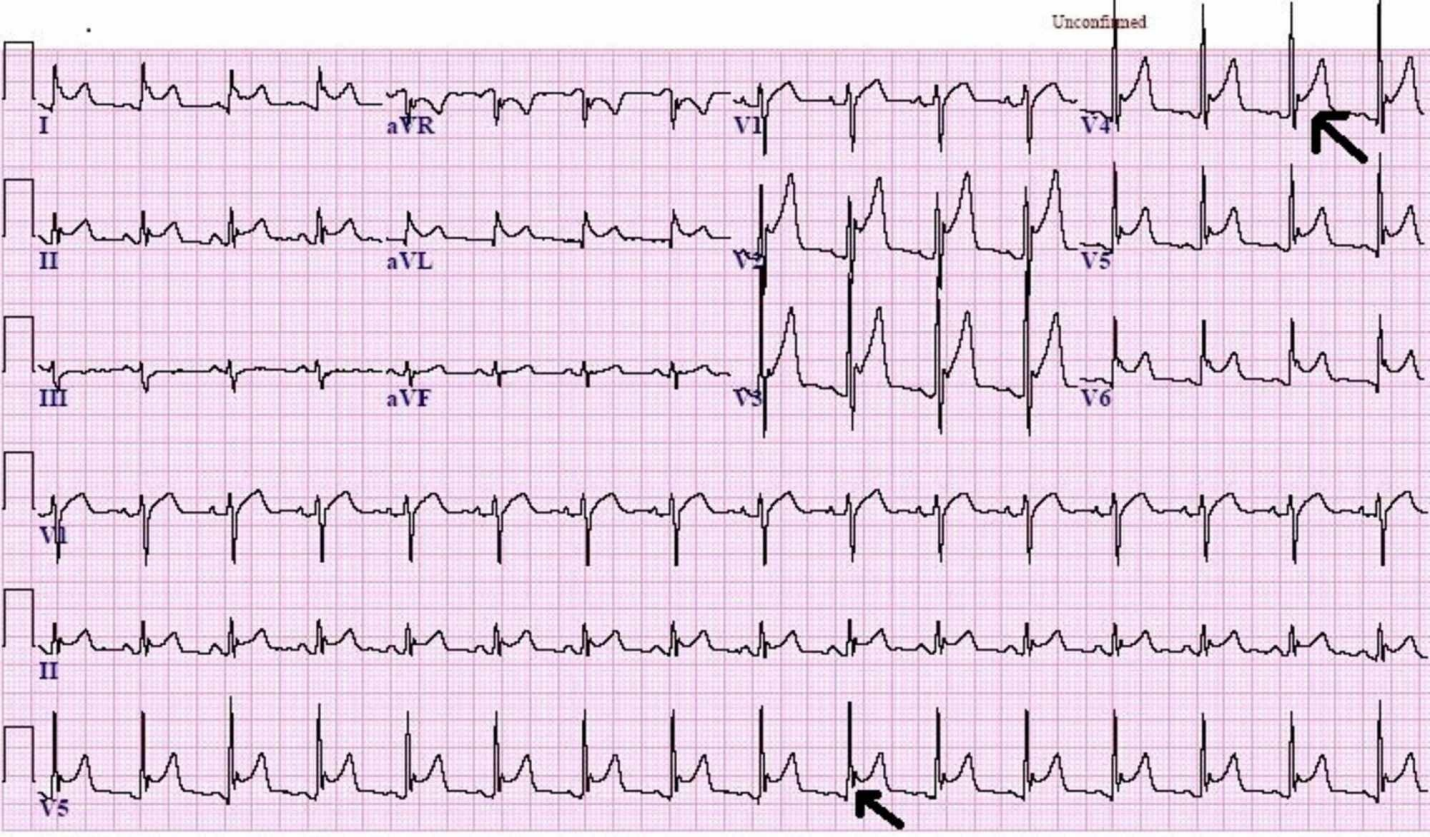
An EKG demonstrating diffuse ST elevation. EKG: Electrocardiography; ST: ST segment.

On the fifth day of admission, a comprehensive physical examination presented normal vital sign findings, except for an oxygen saturation of 87% and a respiratory rate of 26 breaths/min. Interestingly, the continuous electronic vital signs monitor revealed an extensive and diffuse ST-segment elevation finding, which prompted the medical team to order electrocardiography, coronary angiography, and echocardiography. Coronary angiography confirmed a normal supply of blood to the heart and no signs of any arterial obstruction. However, an electrocardiography (EKG) demonstrated diffuse ST elevation (Figure 3), while transthoracic echocardiography indicated an enlarged heart with a marked decrease in ventricular systolic function and an ejection fraction of 20%. Furthermore, elevated levels of troponin (0.29 ng/mL), creatine kinase myocardial band (CK-MB) (20.1 ng/mL) and N-terminal pro-hormone brain natriuretic peptide (NT-proBNP), (BNP; 1,287 pg/mL) were detected. These findings suggested acute myopericarditis, and inotropic support (dobutamine) was thus initiated.

On the sixth and seventh days of hospitalization, the patient’s vital signs indicated a notable improvement. Physical exam findings confirmed bilateral lower extremity pitting edema, a blood pressure of 130/83 mmHg, a heart rate of 88 beats/minute, and an oxygen saturation of 89%. The patient remained on continuous mechanical ventilation and his current medications. Additionally, he was started on a nonsteroidal anti-inflammatory drug (NSAID; indomethacin, 50 mg t.d.s.) on the seventh day of hospitalization to treat acute myopericarditis.

Overall, the patient’s medical condition deteriorated over the following days of his hospitalization, suggesting no dramatic response to his current therapy. His EKGs also continued to demonstrate diffuse ST-segment elevation, and pericardial friction rub was audible upon cardiac auscultation. A cardiac biomarkers laboratory test revealed an increase in troponin (18 ng/mL) and creatine kinase myocardial band (CK-MB) was 14.7 ng/mL, indicating myocardial injury and recurrent myopericarditis. Intravenous methylprednisolone and colchicine were therefore added to the current treatment plan. Repeated echocardiogram exposed a marked decrease in ejection fraction (23%) but no evidence of cardiac tamponade. Chest radiography suggested worsening of the underlying ARDS with bilateral pleural effusion. The patient continued to be managed with aggressive intravenous fluid resuscitation, dobutamine, NSAIDs, antibiotics, and antiviral medications.

## Discussion

Pathophysiology

**Figure 4:**
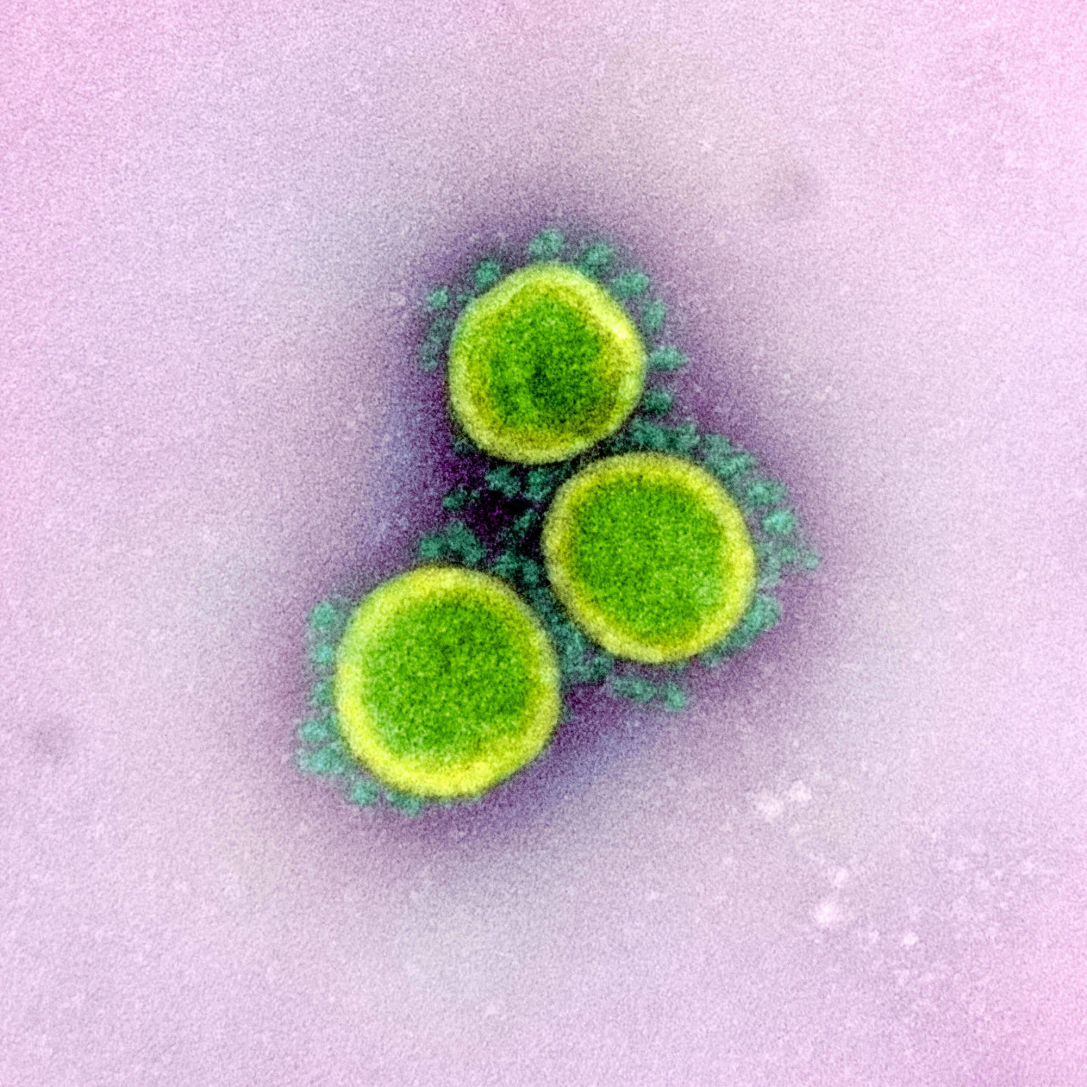
An electronic micrograph depicting the “crown-like” shape of Coronaviridae with the spikes. Image reproduction approved by National Institute of Allergy and Infectious Diseases Credit: NIAID

**Figure 5:**
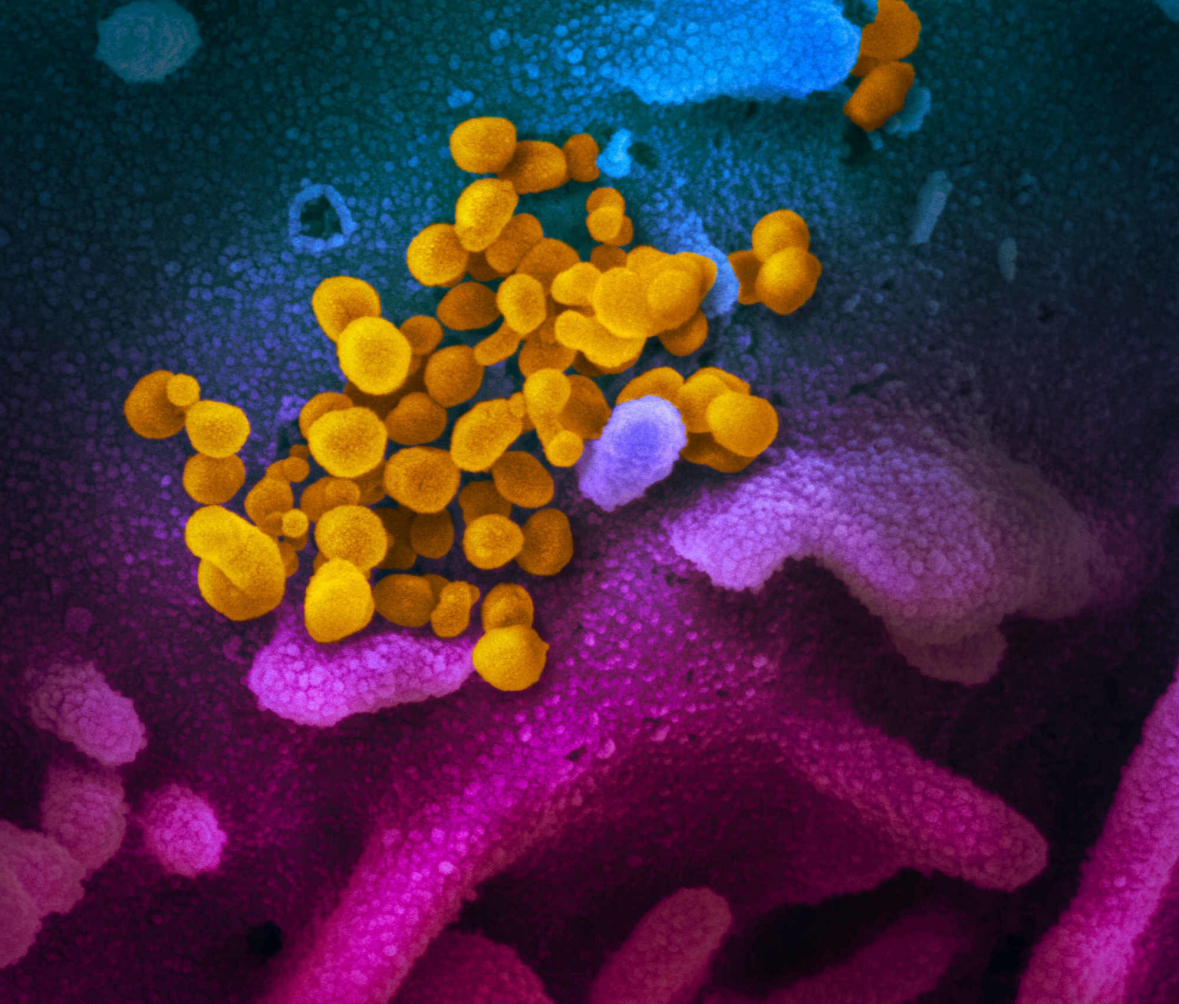
Electron microscope image shows coronavirus (yellow) emerging from the surface of the cell (blue/pink). Image reproduction approved by National Institute of Allergy and Infectious Diseases Credit: NIAID

Coronaviruses (Figures 4, 5) comprise a large family of viruses that cause a wide range of illnesses, from the common cold to more severe respiratory diseases. Found in various species, coronaviruses can produce assorted pathological presentations in each species. All coronavirus subgroups have unique antigenic determinants, culturing requirements, and antigenic cross-reactions between each group, which ensure the virus’s survival and aid in its transmission and proliferation [1].

**Figure 6:**
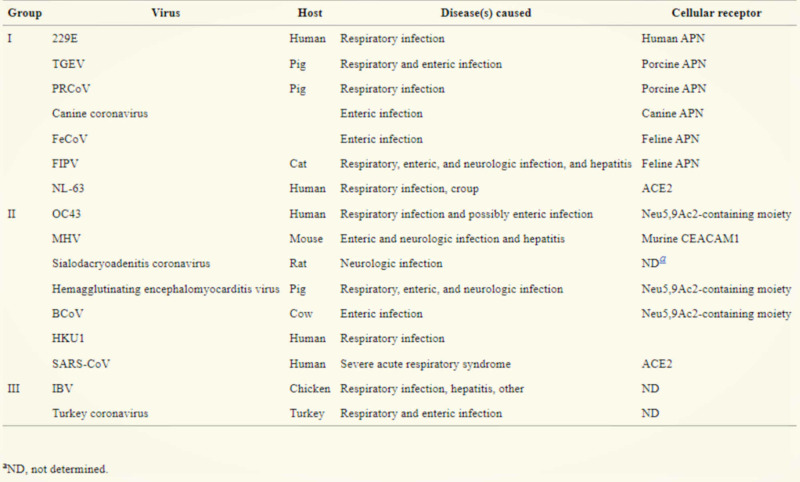
Coronavirus groups, diseases, hosts, and receptors.

The coronavirus family (Figure 6) can be subdivided into three main groups: the first and second are known for their ability to infect humans and pigs, whereas the third is detected primarily in birds [2]. The first two subgroups of the human coronavirus contain four of the six virus strains that infect humans, including 229E, NL63, OC43, and HKU1, all of which are responsible for most coronavirus-related pneumonia and common cold symptoms. Severe acute respiratory syndrome coronavirus (SARS-CoV) and Middle East respiratory syndrome coronavirus (MERS-CoV) are responsible for human-related coronavirus infections less often but cause a more profound and devastating outcome [2].

**Figure 7:**
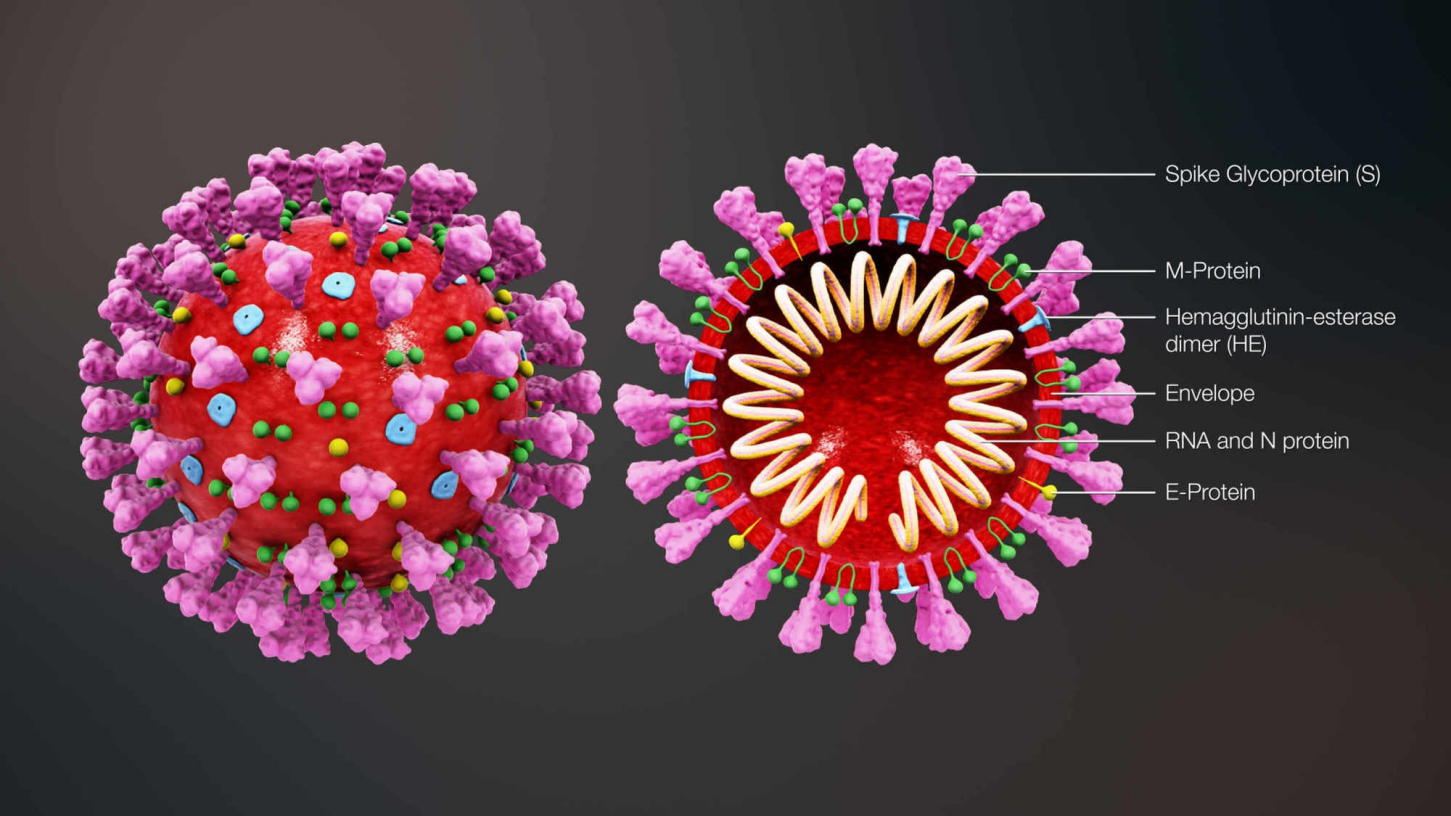
3D animation showing a viral particle contains an internal helical ribonucleic acid (RNA)-protein nucleocapsid surrounded by an envelope containing viral glycoproteins. Nucleocapsid (N) protein is a phosphoprotein that is complexed with genome RNA to form the nucleocapsid. Spike glycoprotein (S) forms the large glycosylated peplomers that are characteristic of coronaviruses. M, the transmembrane protein, is highly hydrophobic and spans the membrane multiple times. E, a membrane-spanning protein, is a minor component of the membrane. Some group II viruses express another glycoprotein, hemagglutinin-esterase (HE), which forms smaller spikes on virions. Image reproduction approved by Scientific Animation Image credit : www.scientificanimations.com

Moreover, coronavirus is an enveloped, positive-stranded RNA about 80-120 mm in diameter. The membrane of the virus consists of four main types of structural proteins. The spike (S) in (Figure 7) contains type I glycoprotein, providing the virus its unique crown shape. This heavily N-linked glycosylated protein is responsible for the fusion and mediation of the host cell receptor’s attachment. In addition, the membrane (M) protein is the most abundant structural protein that surrounds the membrane multiple times; it has an N-terminal ectodomain and a cytoplasmic tail that aids in membrane curvature and nucleocapsid binding. The membrane (E) protein is a highly hydrophobic protein responsible for providing a dense protective barrier that facilitates the assembly and release of virions. Finally, the nucleocapsid (N) protein is a heavily phosphorylated protein responsible for binding to the viral genome and forming the so-called “beads on a string” structure within the viral membrane [2].

**Figure 8:**
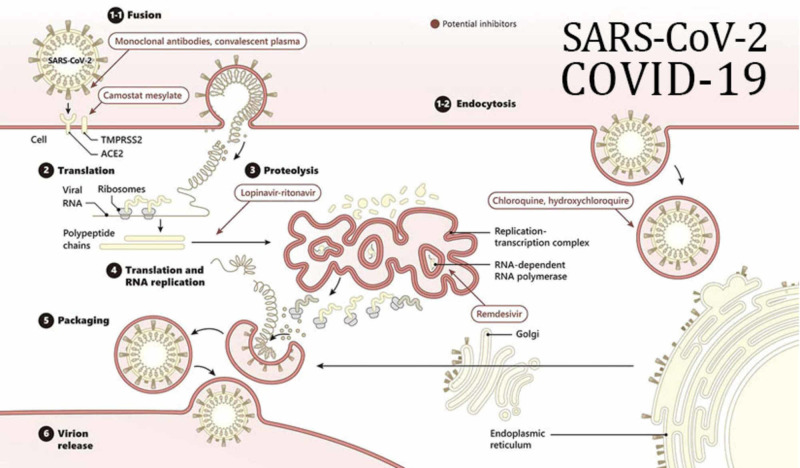
The schematic presentation depicts how the coronavirus attaches to the host’s angiotensin converting enzyme (ACE2) cell receptor. Furthermore, it highlights the different steps in the replication, translation processes and the mechanism of the drugs that have been used recently. These important steps occur with the assistance of the host’s RNA polymerase enzymes. The products are then packaged inside the cellular compartments known as the endoplasmic reticulum and the Golgi apparatus. After this process is completed, the replicated virus utilizes the vesicle trafficking system to undergo exocytosis and infect other host cells. RNA: Ribonucleic acid Image reproduction by Gene Tex Credit: Gene Tex

The coronavirus life cycle (Figure 8) begins when it attaches to the host’s cell receptors via the spike protein [3]. After the attachment process is completed, a series of conformational changes in the spike protein’s structure mediates the fusion between the virus and the cell membrane, which eventually leads to the release of the nucleocapsids into the cell. Once the viral entry process is achieved, the viral genomic RNA begins the translation process by utilizing viral replicas polyproteins pp1a and pp1ab [3]. These sets of proteins are then cleaved into small subunits with the help of viral proteinase enzymes. Polymerase enzymes simultaneously generate several mRNA products through discontinuous transcription to prepare for the translation phase. Viral proteins and genomic RNA are subsequently assembled and packaged into virions in the endoplasmic reticulum and the Golgi apparatus of the cells. Finally, virions are transported out of the cells by utilizing the vesicular transportation system [3].

The virions that escape the local immunological defense system result in a disseminated infection and a cascade of symptoms [4]. The virus particles can enter the blood directly by using fine branching blood vessel networks known as capillaries. The virus then utilizes the body’s natural defense system to transmit respiratory infections to a potential host through droplets produced by exhaling, sneezing, and coughing [4]. Patients infected with coronavirus most often present with various symptoms, including a high spiking fever, a dry cough, a runny nose, and fatigue [4]. Other less common but more severe symptoms include dyspnea, headaches, chest pain, confusion, cyanosis, and restlessness. Patients can also present with nonspecific symptoms, such as epigastric pain, diarrhea, nausea, or palpitations. On rare occasions, coronaviruses can lead to death by directly affecting the cardiovascular system, inducing myopericarditis, altering myocardial contractility, and leading to a drastic reduction in ejection fraction [4]. The first coronavirus subtype that had a destructive effect on the respiratory system is known as severe acute respiratory syndrome (SARS-CoV) which impacted most countries around the world in 2002 [5]. Other form of coronaviruses includes Middle East respiratory syndrome (MERS-CoV) which began in Saudi Arabia and later spread to other countries [6]. Severe acute respiratory coronavirus 2 (COVID-19), which initially began in China, now has been able to spread and expand at an astonishing rate [7]. However, the most common cause of death in patients with COVD-19 infection is ARDS [8].

**Figure 9:**
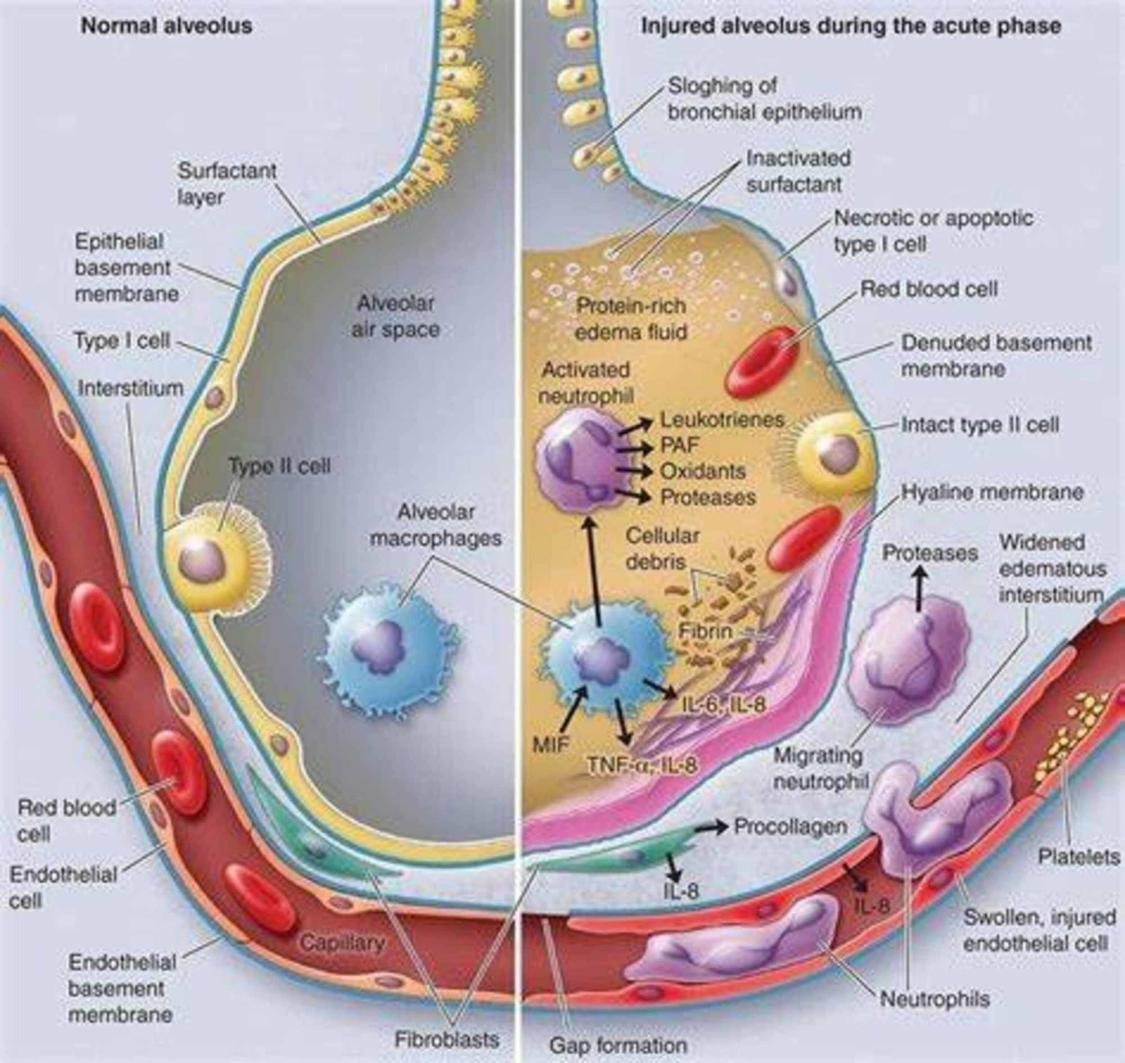
Mechanism of acute respiratory distress syndrome (ARDS) in the alveoli. Image reproduction approved by Health Save Blog Credit: Health Save Blog

A life-threatening lung injury, ARDS is caused by a disruption in the hyaline membrane and diffuse alveolar damage. This vascular disruption results in the leakage of plasma into alveolar spaces and causes the extravasation of the capillaries that surround the alveoli of the lungs (Figure 9). This in turn results in poor oxygenation, tachypnea, tachycardia, dyspnea, and many other respiratory symptoms [9]. Additional risk factors for ARDS include infections, trauma, pancreatitis, sepsis, fat embolism, amniotic fluid embolism, drug overdose, and inhaling toxic substances [9].

Unfortunately, the mortality rate is alarmingly high (globally, yet more than 6,759,210 infected, 395,331 death/ and 2,760,840/recovered) for individuals with coronavirus-induced ARDS due to the complexity of the disease and its difficulty to treat [9]. A minuscule proportion of patients with ARDS will succumb to it on account of respiratory failure alone. Acute respiratory distress syndrome generally causes secondary complications such as sepsis or multiorgan system failure. Although no specific treatments for this disease exist, physicians should provide supportive care therapy to treat it appropriately [9]. Patients with COVID-19-induced ARDS require precise and scrupulous care, which includes the meticulous use of sedative and neuromuscular blocking agents, personalized hemodynamic management, nutritional support, prophylactic measures for nosocomial infections, and deep venous thrombosis prophylaxis and gastrointestinal hemorrhage [9]. As a rule, the first step in managing COVID-19 patients with ARDS is providing proper airway management; patients are first provided with non-invasive ventilation support, such as continuous positive airway pressure (CPAP) or bilevel positive airway pressure (BiPAP) to avoid the complications associated with invasive methods of airway management, such as barotrauma, atelectrauma, and volutrauma. Most patients are expected to require invasive forms of ventilation, such as intubation and mechanical ventilation, to control their respiratory decline and failure. A prone position and extracorporeal membrane oxygenation have recently been advocated as a form of salvage therapy for refractory hypoxemic ARDS [9].

While managing patients with COVID-19 infection is an important component, it is critical to use the appropriate diagnostic measures. The diagnosis of COVID-19 is confirmed with a reverse transcriptase polymerase chain reaction (RT-PCR) diagnostic test, a nuclear-derived method utilized to detect a specific genetic material from a specific pathogen. A sample is collected from parts of the body where the coronavirus generally resides, most commonly the nasopharynx [10]. Other diagnostic methods, including C-reactive protein (CRP) tests, erythrocyte sedimentation rates, complete blood counts, comprehensive metabolic panels, lactate dehydrogenase tests, D-dimer tests, serum albumin tests, creatinine tests, cardiac enzyme tests, partial thrombin and partial thromboplastin time tests, liver function tests, chest X-rays, and chest CT scans are used to not only identify the diagnosis but also predict the progression of the disease and prognostic outcomes [11]. Lastly, many drugs have been used in the treatment of COVID-19 infection including remdesivir, chloroquine, lopiavir, ritonavir, favilavir, hydroxychloroquine, azithromycin, fingolimod and others but none of them has been showing a drastic response. There are varying degrees of quarantine amongst countries [12].

## Conclusions

As this case of a middle-aged male patient demonstrates, COVID-19 infection can cause nonspecific gastrointestinal symptoms, life-threatening ARDS, and myopericarditis. The diagnosis of COVID-19 was confirmed with the help of an RT-PCR. Additionally, abnormal electrocardiography and echocardiography changes validated the diagnosis of myopericarditis. Unlike other coronavirus infections that mainly cause pulmonary infections, this case of coronavirus infection led to profound cardiac damage. Although no specific treatments are currently available, strong evidence suggests that social distancing, quarantine, and isolation produce a drastic reduction in viral transmission, as some of epidemiologic studies have proven.
